# Everyday Activity Science and Engineering Table Setting Dataset

**DOI:** 10.1038/s41597-026-07077-7

**Published:** 2026-05-12

**Authors:** Moritz Meier, Yale Hartmann, Yasmina El Ouahabi, Lars Bredereke, Felix Putze, Tanja Schultz

**Affiliations:** University Bremen, Cognitive Systems Lab (CSL), Bremen, 28359 Germany

**Keywords:** Scientific data, Computational science

## Abstract

Understanding human everyday activity planning and execution is crucial to inform cognition-enabled robots and systems. In this paper, we describe the design, collection, validation, and dissemination of the Everyday Activity Science and Engineering Table Setting Dataset (**EASE-TSD**). EASE-TSD is a dataset of multimodal high-dimensional biosignals synchronously recorded from human subjects who are setting a table in a controlled laboratory setup. Data from 78 sessions are available, each recorded during six table-setting trials in which we capture the planning and execution of human behavior using eight synchronized biosignal streams: marker-based motion capturing, environmental and first-person video cameras, eye-tracking, electromyography, electrodermal activity, acceleration, microphones, and electroencephalography. Participants were instructed to think aloud concurrently and retrospectively to explain and comment on their table-setting actions and the corresponding cognitive processes. EASE-TSD is annotated with a 3-level annotation schema containing phases, activities, motions, and interacted objects. Additionally, the think-aloud (TA) protocols are annotated using TA codes. After recording, the EASE-TSD data undergo semi-automatic labeling, post-processing, and analysis procedures, leveraging latest biosignal processing and machine learning methods.

## Background & Summary

Recent advancements in machine learning and robotics pave the way for a promising future with cognitive-enabled robotic assistants that can interact with and support humans in their everyday lives^1,2^. Kitchen scenarios are some of the most frequently studied activities due to their well-defined locations, high complexity, and underspecified instructions, which often require multiple sensors to master. Common scenarios include meal preparation^3–10^, table setting^11–13^, and human sensory motion understanding^14–18^. In their studies, authors have used head-mounted cameras^4,9,12,19^, stationary cameras, sensors placed in the environment^3,5– 9,11,12,20,21^, as well as sensors attached to the participants^3,8,9,18,22,23^. Numerous advantages are outlined for interpreting human needs through the synchronous capture of various biosignals, providing a comprehensive understanding and holistic view of users, to subsequently enabling biosignal-adaptive systems and robots to be tailored to the interpreted individual users needs^24–26^.

Most published studies either focus on a small number of participants to get a holistic view of a participant, target the human planning/execution process in particular, or emphasize large-scale data collections with many participants but less detailed focus. To the best of our knowledge, it is uncommon for a single study to address all three aspects.

**EASE-TSD** is such a study that tackles all three aspects in one dataset. It provides a comprehensive view of the participants with eight different sensor types (totaling 22 sensor devices), gives insights into the participants’ planning processes derived from brain activity recordings, as well as from concurrent and retrospective think-alouds (TA), and it covers a significant number of subjects with 78 sessions (of which 50 are thoroughly analyzed in this paper). EASE-TSD includes multiple synchronized biosignals recorded in the Biosignals Acquisition Space and Environment (BASE)^12^ at CSL, a member of the EASE collaborative research center (EASE CRC, http://ease-crc.org). As elaborated later in this paper, the data is post-processed and annotated at three levels of granularity (action, phase, and motion) and transcribed using the participants’ think-alouds and retrospection protocols, following the methodology established in our previous work^27,28^.

**Fig. 1:**
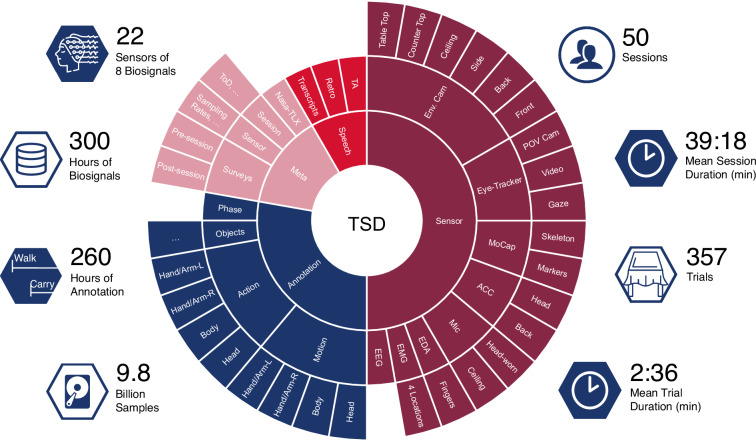
Dataset Overview. The left-hand side lists key statistics, including the number of sensors, accumulated sensor recordings, accumulated annotations across tiers, and the number of vector samples. The middle sunburst diagram depicts all information sources, including sensors (dark red), annotation levels (blue), meta-information (pink), and speech (red). The right-hand side lists the number of sessions and trials, as well as their respective mean durations.

Figure 1 presents key parameters of EASE-TSD in one plot (Icons with permission from Cisco and Flaticon), i.e. all sensors, annotation tiers, and meta information. Furthermore, key statistics, like the mean session duration, the accumulated duration of sensor signal data, and the accumulated duration across annotation tiers, are depicted.

Table 1 provides a comprehensive summary of the key parameters of related datasets on everyday activities. These datasets focus on some combination of (1) a large number of participants, (2) a large number of everyday activities, (3) a large number of sensors, or (4) a high recording quality. **EASE-TSD** uniquely combines all four aspects in one database. The number of activity classes is in the middle range at 33, while the number of participants is at the upper end with 49. The number of sensors and RGB camera perspectives is higher compared to most other datasets. The unique selling point of EASE-TSD is its rich set of everyday activities synchronously recorded in terms high-dimensional biosignals, which include Electroencephalography (EEG), Electrodermal Activity (EDA), think-aloud and retrospection in a kitchen scenario. Furthermore, only few datasets consider multiple tiers of granularity in annotation, which provide crucial information for understanding the participants’ planning process.

**Table 1:** Key parameters of common activity datasets compared to EASE-TSD.

Dataset	#Class	#Subj	#RGB	TA/Retro/Tiers	Environment/Activities	Modalities
TUM Kitchen^11^	10+	—	4	✗/✗/*✓*	Table setting	RGB, 3D Joints, RFID, Reed switches
CMU-MMAC^59^	5	43	6	✗/✗/✗	Kitchen activities	RGB, Audio, 3D Joints, IMU
MSR-Action3D^17^	20	10	1	✗/✗/✗	Exercise/arm motions	Depth, 3D Joints
OPPORTUNITY^60^	9+	12	3	✗/✗/*✓*	Daily actions	IMU, Audio, RGB, RFID, UWB Localization, Reed switches
UCF101^61^	101	—	1	✗/✗/✗	Daily actions	RGB
Fine Grained Cooking^6^	65	12	1	✗/✗/✗	Meal preparation	RGB
UT-Kinect^16^	10	10	1	✗/✗/✗	Daily/arm motions	Depth, 3D Joints
CAD-120^62^	10	4	1	✗/✗/✗	Kitchen activities	RGB, Depth
Multiview 3D Event^63^	8	8	3	✗/✗/✗	Daily actions	RGB, Depth, 3D Joints
Complex Cooking^5^	17	27	1	✗/✗/✗	Meal preparation	RGB, Depth, ACC
JHU Table Setting^13^	—	—	1	✗/✗/✗	Images of set tables	RGB
Breakfast^10^	10	52	3-5	✗/✗/*✓*	Meal preparation	RGB
Office Activity^64^	20	10	3	✗/✗/✗	Office activities	RGB, Depth, 3D Joints
Watch’n’Patch^65^	21	7	1	✗/✗/✗	Daily actions	RGB, Depth, 3D Joints
UTD-MHAD^66^	27	8	1	✗/✗/✗	Exercise/arm motions	RGB, Depth, 3D Joints, IMU
Egocentric Activity^19^	20	—	1	✗/✗/✗	Daily/office/exercise	RGB, IMU
Something Something^67^	174	1133	1	✗/✗/✗	Daily actions	RGB
Daily Intention^68^	10	12	1	✗/✗/✗	Daily actions	RGB, ACC
Stanford ECM^23^	24	10	1	✗/✗/✗	Physical activities	RGB, Heart Rate, ACC
MMAct^22^	37	20	5	✗/✗/✗	Daily actions	RGB, IMU (phone & watch), Wi-Fi, Barometer
NTU RGB+D^21^	120	106	3	✗/✗/✗	Daily actions	RGB, Depth, IR, 3D Joints
MoCA^3^	20	—	3	✗/✗/✗	Meal preparation	RGB, 3D Joints
Epic Kitchens^4^	397+	37	1	✗/*✓*/✗	Kitchen activities	RGB
CSL-SHARE^18^	21	20	—	✗/✗/✗	Daily actions	IMU, EMG, Goniometer, Audio
Kinetics-700^20^	700	—	1	✗/✗/✗	Daily actions	RGB
ActionSense^9^	20	10+	7	✗/✗/✗	Kitchen activities	RGB, Depth, 3D Joints, Gaze, IMU, EMG, Tactile, Audio
**EASE TSD**	**33+**	49+	7	*✓*/*✓*/*✓*	**Table setting**	**RGB, 3D Joints, Gaze, ACC, EMG, EDA, Audio, EEG**

The table is adapted and extended from Del Preto *et al*.^9^. TA= Think-Alouds, Retro=Retrospection, RGB=RGB-Camera, IMU=Inertial Measurement Unit, ACC=Acceleration, RFID=Radio-Frequency Identification, UWB=Ultra WideBand Localization, IR=Infrared, EMG=Electromyography, EDA=Electrodermal Activity, EEG=Electroencephalography. Epic Kitchens has a very large number of classes as it uses noun and verb phrases as classes.

The multimodal recordings in **EASE-TSD** can be used to investigate multiple facets, including classical machine learning tasks like human activity recognition, (multimodal) sensor fusion/binding, and human/object pose estimation. Furthermore, it supports insights into human planning of everyday activities, such as table setting, with cognition-related modalities like EEG and eye tracking. Finally, the dataset is accessible to cognitive robots, for instance, via Narrative-Enabled Episodic Memories (NEEMs)^14,29^.

This paper is structured into four main sections: Methods, Data Record, Technical Validation, and Code Availability The Methods section describes the complete data processing including recording, annotation, and post-processing. The recording spans the quantitative data readings from the different sensors during the performed task, the NASA Task Load Index, and the qualitative data from mental insights via think-aloud protocols and questionnaires. The **EASE-TSD** data collection comprised 100 participants. The data of 78 participants, who consented to data publication, is freely available at https://osf.io/rbyfk, accompanied by comprehensive documentation and annotation. The analyses presented in this report are based on the first 50 sessions. The Data Record section details the available data, including file structure, formats, and content. The Technical Validation focuses on identifying issues such as missing files, on analyzing activity durations, and on developing machine learning baselines. At the time of publication, the dataset has been used by several partners in the EASE project, in various bachelor and master theses, and in Machine Learning competitions (namely the Bremen Big Data Challenges 2018, 2020 and 2022: https://bbdc.csl.uni-bremen.de/). The Code Availability provides a list of tools developed, their intended purposes, and their access locations. In summary, this article introduces our **EASE Table Setting Dataset (EASE-TSD)**, details its recording and processing methods, and presents its initial analyzes.

## Methods

The dataset is created in three main stages: dataset collection, post-processing, and annotation. The Data Collection process is designed to capture the participants’ activities during the table-setting task, including their movements, speech, and cognitive processes. The data collection comprises the experiment procedure, sensor setup, and technical architecture. The sensor setup involves placing the sensors on the participants in a specific order to ensure accurate data collection. The experiment procedure includes a series of trials in which the participants set a table in different configurations while thinking aloud. The Post-processing stage involves converting the raw data into a suitable format for convenient parallel processing of multiple modalities from many or all sessions. For example, this involves cutting signals to a common length, correcting any timing issues that occurred during recording, and subsequently checking the technical validity of the data. The Annotation process involves transcribing the participants’ speech and annotating their activities during trials. The human activities were annotated using multiple *tiers* with fine-grained labels. For this purpose we extended the ELAN tool^30^ such that we introduced hierarchically inspired parallel annotation tiers, such that some tiers coarsely describe activities while others are used to annotate two parallel motions. More details are provided in Annotation.

All custom code, if not indicated otherwise, is available at the public gitlab repository: https://gitlab.csl.uni-bremen.de/ease-public/

### Data Collection

The data collection stage is the most time-consuming but also the most crucial stage. The aim of this data collection is to capture the participants’ activities during a table-setting task and to get a holistic view of the participants’ behavior, including their movements, speech, and cognitive processes. Figure 1 gives an overview of the various sensors used. Care needs to be taken in the technical orchestration as well as the sensor setup. The recording process takes approximately 3:20h per participant and is conducted in a controlled laboratory environment. The overall process is described in the following three sections: Experiment Procedure, Sensor Setup, and Technical Architecture. Participants were recruited through advertisements on social media, physical and digital university blackboards, lecture announcements, and other channels which allowed such announcements. To avoid strong biases in the data, we did not formulate strong exclusion criteria: participation was possible as long as the person felt able to set the table in a typical German kitchen setting and had a good command of German or English. Participants were informed about appropriate clothing and hair style compatible with the EEG gel application and the tracking suit for motion capture.

#### Ethics & Consent

The University of Bremen granted ethical approvals for this study on 7 April 2017 and 2 March 2021 under the identifier 06-3. Participants give written consent on the participation in the study and in the scientific use of their data. For the latter, we offered them two choices: They could consent to the publication of the data including video and audio data which would reveal their identity. Alternatively, they could participate in the study with the access to the data staying limited to research purposes within the project. Before deciding on giving consent with either option, participants had the opportunity to read the provided information material, outlining the nature of the experiment, the employed sensors, and the consequences of the two data handling options (for example, the chance of being identified and potential limits to the ability for retroactive deletion). This process was developed in cooperation with legal scholar of the universities ethics committee to find a balance between availability of raw data for scientific validity and concerns of privacy and data agency. The consent form and the information material can be found in the data repository.

#### Experiment Procedure

**Fig. 2:**
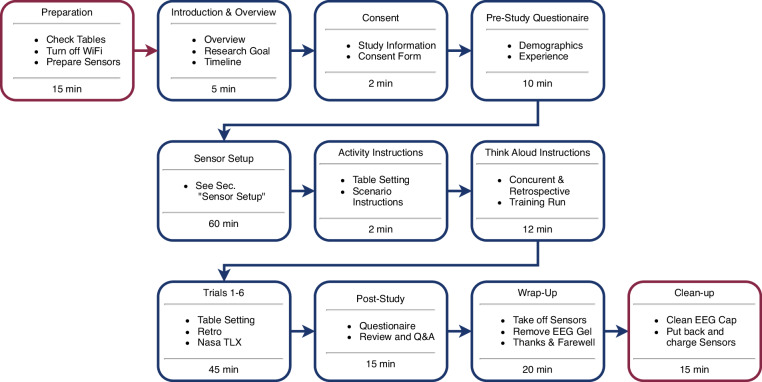
Experiment Protocol. Blue boxes mark sections with participant interaction, while red boxes mark sections of preparation for the researchers only.

All recording sessions follow the same protocol, starting with the preparation of the lab and sensors, and ending with cleaning, as shown in Fig. 2. Generally, no two sessions are recorded with the same participant. The only exceptions to this rule are sessions 63 and 64, which are recordings of the same participant but are marked as separate sessions due to technical considerations. Furthermore, some participants were willing to record more than the six planned trials, as shown in Fig. 2.

The experiment setup ensures that all sensors are ready and any interference is minimized to ensure the best possible signal quality. This preparation corresponds to the first step in Fig. 2. The sensor preparation includes charging batteries and collecting all 22 sensors from their storage. The interference reduction includes turning off or covering all unused wireless devices, e.g., WiFi and infrared emitters, e.g., VR equipment or outside-facing windows. Furthermore, it is ensured that the tables are in the exact same location via markers on the floor and that the cameras are correctly pointed inside the BASE.

Welcoming the participant, explaining the experiment, and signing the consent form are subject to the next step. The preexperiment questionnaire (assessing biographic information and background) is answered by the participants. The questionnaire includes a question for formal table setting prior knowledge, for example due to work in the gastronomic sector.

The sensor setup is carried out as described in section Sensor Setup and is completed by two resting EEG recordings for baseline measurements. For this recording, the participant sits, first with eyes open, and then closed for two minutes each, with no disturbances. The resting EEG is followed by a test of thoughts aloud (often noted as t0 in the data records) in which the participants remove the dining table from multiple items. The participants unset the table instead of setting it to avoid performing a trial configuration before the planned recordings begin.

The six table-setting trials performed form the core of the experiment. Each of the six configurations is performed in random order. The configurations mimic different typical table-setting scenarios and are as follows: informal breakfast for two/four people and informal/formal lunch for two/four people. The participants are informed about the scenario and the number of seats before each trial via the projector within the BASE. They were instructed to take any selection of items from the side table and place them how they feel fit for the respective scenario. This meant that it was in their discretion to decide which selection an layout was appropriate, for the different scenarios (e.g., formal vs. informal). There were no further restrictions on the table setting process or the final table layout. We include all instruction documents as they were shown on the projector screen to the participants in the supplementary material of the data repository. These give the exact information provided to participants for performing the different task variations.

Each trial consists of six steps: **(1)** Trial condition speak-out for better memorization and to avoid interaction with the researcher during the recording **(2)** the participant takes up the T-Position crucial for motion capture marker calibration, **(3)** the participant indicates readiness and repeats the trial conditions **(4)** the researcher triggers the recording, the lights turn on and the participant performs the table setting task **(5)** the participant indicates that the task is finished, then adopts the T-position and faces the projector screen **(6)** a third-person perspective video of the just performed task is shown and the participants performs the retrospective think-aloud protocol of their actions **(7)** lastly the participant fills out the NASA TLX while the researcher resets the table and counter. This process repeats for all six trial configurations. During each table setting, the participants may choose any number of items from the side table and set them in any configuration on the dining table. Once they deem the task finished, they end the trial. Participants may choose to perform additional trials if they do wish so. In this case, they will repeat previous settings. Three of the six trials are randomly selected as think-aloud (TA) trials, in which the participant describes verbally what s/he is doing, while s/he is doing it. Using Automatic Speech Recognition (ASR), transcriptions are created that form Think-aloud protocols, which are valuable tools in studying the correlation between verbal output and task completion. The other three trials are referred to as silent trials. Since speech produced during TA might have an impact on other sensor recordings, it is vital to balance data quality with minimal interference. Furthermore, accurate semantic descriptions are generated, and time limitations are considered.

The Post-Session phase in Fig. 2 includes a post-experiment questionnaire, a debriefing and a final interview. The post-experiment questionnaire assesses the participants’ experience and satisfaction with the experiment. The debriefing answers and records any questions, comments, or feedback the participants may have. The wrap-up and clean-up complete the experiment.

#### Sensor Setup

EASE-TSD recordings include a variety of biosignals: electromyography (EMG) and electrodermal activity (EDA) from the lower right arm, acceleration (ACC) from the back and head, full-body marker-based motion tracking, mobile electroencephalography (EEG), think-aloud speech recorded from a head-worn microphone, mobile eye-tracking, first-person video, environment audio recorded from a ceiling microphone, and six environment RGB cameras as displayed in Fig. 1 and Table 2. Each sensor records an essential aspect of the participant’s table-setting process. Due to the large number of sensors, the order in which the sensors are set up is crucial. The following introduction explains this order along with practical lessons learned during setup. Detailed documentation, including video guides, will be added to the dataset and released at the time of publication.

**Table 2:** Sensor Setup Overview.

#	Sensor	Vendor/Model	Samplerate	Channels	Resolution	Location
1	Mic. - Env.	Audio-Technica Pro45	44.1 kHz	Mono	16 bit	Ceiling
6	Camera - Env.	Logitech C930	30 Hz		720p	Back, Ceiling, Counter-Top,Front, Table-Side, Table-Top
8	EMG	PLUX	0.6/1 kHz	Bipolar	16 bit	Flexor Carpi Ulnaris and Radialis, Flexor Digitorum Superficialis, Extensor Carpi Radialis (left and right arm)
1	ACC	PLUX	0.6/1 kHz	3-Axis	16 bit	Participants back
1	EDA	PLUX	0.6/1 kHz		16 bit	Index and Middle Finger
1	Motion Capture	OptiTrack	150 Hz	49 markers		Participant worn suit
1	EEG	g.Nautilus	500 Hz	16 Channel	24 bit	Participant worn cap
1	ACC	g.Nautilus	500 Hz	3-Axis	24 bit	Back of EEG Cap
1	Mic. - TA	Sennheiser HSP 4	44.1 kHz	Mono	16 bit	Participant head-worn
1	Eye tracker	Pupil Core	120 Hz	2/3D Gaze	240p	Headworn
1	Camera - POV	Pupil Core	30 Hz		1080p/720p	Eyetracker

The Audio-Technica Pro45 **environment microphone** records ambient noise (16-bit resolution at 44.1 kHz) and is suspended at a distance of approximately 3.5 m above the recording environment. It provides crucial information about precise timings in item placements or cutlery collisions. Especially in combination with the video, it offers a complete scene understanding. The microphone does not require extra setup and is turned on once the recordings start.

Six strategically placed **environment RGB Cameras** provide a full view of the experiment area: three side views, two tabletop views, and one ceiling view. The videos can be used to track participants across the room, recognize their actions, and identify interacted items. The videos are also used for annotation in the post-processing step (see Section Post-processing). The files are recorded as MJPG and later converted to H.264 encoded videos. At the beginning of the experiment, any accidentally dislodged camera is refocused. Then, the cameras are turned on for the experiment.

Four bipolar PLUX (https://www.pluxbiosignals.com/) **electromyography** (EMG) sensors capture the muscles of the lower arms (as listed in Table 2) with the ground electrode placed on the participant’s collar bone. Placement followed the positions used by^31^ for capturing hand movement and followed the general reccomendations by the SENIAM project^32^ (in terms of inter-electrode distance, skin preparation, electrode and amplifier settings, etc.). The EMG signal complements motion tracking by providing detailed recording of finger movements when grasping, carrying and releasing. These details are shown by changes in EMG amplitude indicating muscle contraction. Due to the Electromechanical Delay (EMD) of roughly 10 ms^33,34^, EMG signals capture motion intent, such as grasp intent^35^, before any force is measurable and have proven themselves in human activity recognition studies^31,36^. The EMG electrodes are placed first of all sensors, as the motion tracking suit will be worn over them. The EMG and acceleration signals are recorded with hardware-synced PLUX biosignal hubs fastened to the trousers of the motion capture suit and streamed via Bluetooth to a recording computer.

The **electrodermal activity sensor** (EDA) captures skin conductivity and is placed on the palm of the participants’ non-dominant hand, according to the placement guidelines of the device manufacturer. It indicates sweating and can thus provide insights into the activity of the sympathetic nervous system. The EDA uses the HW-synced PLUX-Hubs, and due to technical reasons, was abandoned for later sessions (see Fig. 6).

A 3-axial **accelerometer** (ACC) captures the participant’s body trunk movement. Its high temporal resolution adds information to the motion tracking system while also providing redundancy. The accelerometer is attached to the suspenders in the participant’s back, as close to the *BackTop* motion capture marker (roughly between vertebrate C7 and T1) as possible. Exact positioning depends on the fit and shift of the suspenders the sensor is attached to. The data is streamed from the hub via Bluetooth to a receiver computer, from where it is relayed to the recording computer.

The OptiTrack **Motion Tracking System** comprises nine active infrared cameras (OptiTrack Prime 13 W) fixed in the BASE environment and a velcro suit with 49 attached reflective markers. The tracking markers for the head were attached to the EEG cap at stable positions (the exact position is documented in a picture in the data respository). Motion tracking allows precise tracking of the complete skeleton, including all extremities, the head, and the fingers. The latter precision suffers slightly due to the distance of two meters to the nearest camera. Skeleton tracking can be used to model activities such as ground truth for pose estimation from the environment cameras or for EMG and EEG motion intent studies. The cameras and recording software are started before the experiment. The velcro suit is carefully put on by the participant, with help from a researcher, so as not to loosen the EMG, EDA and ACC sensors. Some markers are added to special gloves, the laptop backpack, and the EEG cap. All markers are checked and adjusted, and the skeleton is created before recordings start. Furthermore, the participant enters the T-Pose at the start of each trial. The T-Pose involves raising the arms to the side with the feet hip-far apart, forming the letter T, and ensuring a good calibration during the experiment.

The g.Nautilus Research is a mobile and wireless **electroencephalographic** system using active gel electrodes. It is configured to use 16 channels of the International 10-10 system (Fp1, Fp2, F3, Fz, F4, T7, C3, Cz, C4, T8, P3, Pz, P4, PO7, Oz, PO8), a ground electrode (located at AFz), and a reference clip attached to the right ear lobe. The EEG signal aims to capture information about the mental state, attention, and focus and may give insight into the planning process, especially in combination with the think-aloud and retrospection protocols. The signal also provides insights into the sensorimotor system and may be used to predict activities and motions captured by, for instance, the motion tracking system. The EEG signals should be combined with the included accelerometer and the eye tracker to identify and remove head and eye movement artifacts and combine fixations with EEG data^37^. The EEG cap size is *tested* before anything else, as switching electrodes to a different size adds half an hour to the recordings. The EEG cap is *put on* after the motion capture suit, and the gel is only applied *after* the eye tracker is calibrated, and the first recording is about to start to minimize drying out and any cap misalignment due to later sensor placements. The impedance is kept below 100 *Ω* as best as possible, and the data is streamed via the Nautilus hardware to the receiving computer.

The **head-worn microphone** (Sennheiser HSP 4) records at 44.1 kHz (see Table 2). It is used for the think-aloud and retrospective protocols, where participants describe their own activity during the trial (concurrent think-aloud) or just after, commenting on themselves in a video (retrospective think-aloud). The reports grant insight into the participant’s cognitive processes, manipulation techniques, task completion conditions, and action decision-making^38^, as well as provide a source for activity labeling^28^. The microphone is placed around the neck, just in front of the participant’s mouth, and is carefully placed so as not to interfere with the EEG and to leave space for the eye tracker. The microphone also records during non-think-aloud trials and acts as a second environment microphone.

The PubilLabs **eye tracker** consists of two cameras capturing pupil movement and dilation, mounted on a frame below the eyes and one 100-degree wide-angle outward-facing first-person camera. The eye tracker is used to track participants’ gaze during the table-setting trials and can benefit the planning or mental state models as well as understanding when vision is required for precise motion. The eye tracker is added after the microphone and then calibrated. The gaze confidence is included in the exported files. The eye tracker is moved as little as possible while the EEG electrode gel is applied. The recording laptop is placed in a custom-made backpack and connected via USB. After approximately an hour, the participants are ready for the experiment. While the number of sensors require this substantial setup time, we carefully chose the components to be as little impedimental as possible. The eye tracker device has a weight of just 100g, comparable to a pair of glasses, the EEG headset (including the amplifier) has a weight of 240g, comparable to a light bicycle helmet. The tracking suit is made out of light spandex cloth and the tracking markers. These choices allowed participants to move freely and naturally in the scene.

#### Technical Architecture

**Fig. 3:**
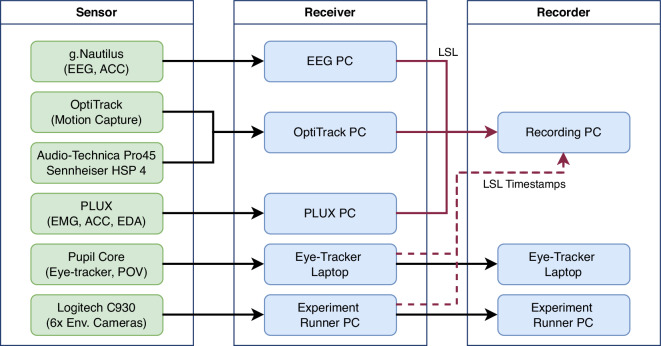
Sensor and PC Architecture. Sensors in green, computers in blue. Datastreams in black, and the Lab Streaming Layer (LSL) streams in red. LSL streams with only timestamps use dotted lines.

The technical architecture is crucial for recording quality with regard to the chosen methodology and experiment procedure. It includes two main components: (1) the experiment runner and (2) the different sensors and their recording hard- and software. The experiment runner is responsible for orchestrating the recording, the lighting, the projector images, and more. It gives the researcher important cues about the trial’s configuration and sensor status, and it is responsible for showing the video of the participant’s table setting during retrospection. Therefore, it is crucial for the experiment runner computer to have direct access to the video files without any network lag. Accordingly, as depicted in Fig. 3, the experiment runner is connected to the environment cameras and the projector. Before each trial, all sensor recordings are stopped and restarted for state management reasons in some libraries. Figure 3 shows the wiring for all sensors. It depicts three main stages: the sensor itself, the (sometimes proprietary) receiver, and the relay to a recording computer.

#### Synchronization

Lab Streaming Layer (LSL)^39^ is a networked middleware to stream, receive, synchronize, and record heterogeneous data streams and markers from different sources. LSL allows to synchronize the clocks between the different recording computers. Due to network bandwidth limitations, the video sensors are not relayed via LSL. However, the timestamps of all frames are captured via LSL to ensure synchronization between all sensor samples. Note that a minimal delay between sensor recording and the final timestamp may occur due to buffering on the receiver side. This is most notable with Bluetooth-based sensors like the PLUX sensors or video-based sensors with occasional USB buffering. Where possible, this was corrected in post-processing (see Post-processing).

As annotation is performed on the basis of the 30 fps video and this modality is also most prone to delays due to frame buffering, the temporal precision we can expect from the recorded signals is at most 1/30*s* ≈ 33.3*ms*. Through an external stimulus visible in all different sensors (a forceful clap), we verified that latency between below 0.4s for the different video streams. By inspecting the synchronization logs of the LSL streams, we found that time stamp jitter is on average 0.98ms for different streams and as the clock differences are handled by LSL, this is the maximum uncorrected latency we can assume between different sensors from synchronization. An implication of these observations is that video-based annotations might be not precise enough for annotating small-scale physiological responses to individual stimuli, such as an EEG event related potential triggered by an event in the scene (which one would expect to occur in the range of hundreds of milliseconds after the stimulus); however, other types of signal analysis which consider segments in the magnitude of multiple seconds, such as determining mental workload from EEG, this temporal precision is sufficient.

### Post-processing

All data, including MJPEGs and LSL streams, is initially stored raw and requires multiple preprocessing steps before final visualization, annotation, and archiving.

First, the frame timestamps of the environment cameras are fixed. Due to buffering in the USB connection, the webcam frames sometimes would be delayed and released at once. These instances are found, and the frames are spread equidistant to match the 30 Hz frame rate. On several occasions, frames were dropped entirely from the buffer. These are fixed by duplicating the last frame. Second, the different modalities’ time-series are cut to the length of the shortest valid signal. The recording lengths vary due to the necessity of multiple parallel recording computers and the consecutive starts of the sensors. However, due to the given LSL (https://github.com/sccn/labstreaminglayer) timestamps, the same-length trimming and alignment are straightforward, resulting in synchronized trial recordings. Third, ASR Whisper^40^ is applied to automatically transcribe the think-aloud speech, resulting in the TA protocols. The small Whisper model and the Whisper-timestamp library^41^ are used to create timestamps for the transcriptions. In addition to the automatic transcripts, we also provide manually corrected transcriptions, available in the manual files.

Fourth is motion capture matching. Due to technical issues, the motion capture stream relay into LSL sometimes did not stream the OptiTrack skeleton. In these cases, we matched the locally stored motion capture markers (stored by the proprietary software) with the LSL-stored markers using the lowest Euclidean distance and a manual threshold. Fifth, the channel names and sensor sampling frequencies are double-checked and estimated using the existing timestamps, as adjustments were made for necessary setup tweaks during the recording years. For a full list of sensor sampling rates per trial, please check the metafile of each sensor. The reported sampling rate is checked automatically using the mean difference between timestamps and plotting the difference histograms.

Sixth, an ”aligned" time series is created based on the timestamps and known sampling rates. The alignment adjusts each LSL timestamp to the closest possible timestamp, assuming the first sample is created at *t*_0_, and all subsequent samples are a multiple of the sensor’s sampling rate. This allows for easy merging of the different modalities using standard SQL merge queries over this column. Since the least common multiple of all sampling rates (10 Hz) is quite small, merging might still create a lot of missing values and large file sizes. Lastly, tabular data is converted into Apache Parquet, video data into MP4 (H.264 codec), and audio into WAV files for convenient access. Those sessions, for which participants consented to internal use only are excluded from the public dataset. The formatting of the final data records is described in detail in Data Record.

Currently, we are working on (i) including automatic tracking of all involved objects utilizing the six environment cameras, (ii) training recognition models for automatic initial annotation with human verification, and (iii) automatic logging of automatic Narrative Enabled Episodic Memories (NEEMs). These files will be made public as soon as they become available.

### Signal Quality

To quantify the signal quality of the recordings, we estimated signal-to-noise (SNR) ratios for a selection of signals. For the EMG signal, we calculated the average signal energy as sum-of-squares for all instances of “grasp” (as signal) and for the resting period in which no object was grasped. The resulting SNR was 18.4 dB, which is in line with other reports of grasping-related EMG^42^. For speech, we performed a similar calculation using the silent audio during the resting period as noise and the speaking segment (as determined by the Pyannote (https://github.com/pyannote/pyannote-audio) speech diarization toolkit) as signal. This resulted in the values 34.92 dB and 16.15 dB for the close-talking microphone and the ceiling microphone, respectively. While these numbers are relatively low for pure speech analysis, it is an expected effect due to the frequent non-speech sounds accompanying the table setting situation. Finally, for EEG we calculated SNR based on the Berger effect: For the resting recordings, we calculated the signal energy in the *α*-band (8-13 Hz) and contrasted this with the signal energy in the *θ*- (4-8 Hz) and *β*-band (13-30 Hz), as we would expect a strong *α* synchronization in a resting state. As the data set contains resting segments with eyes open and eyes closed, we could compare those to see the Berger effect of increased *α* synchronization with closed eyes (and thus no visual intake), reflecting a resting rhythm of the visual cortex. Reflecting this, the SNR was 2.31 dB for the eyes open condition and 5.34 Hz for the eyes closed condition. As EEG is a very noise signal, such comparably low SNR is expected and indicates usable neural data.

### Annotation

Annotation is a crucial part of any dataset creation. It aims for the data to be labeled and structured in a way that is useful for analysis. In the EASE-TSD dataset, the annotation is three-fold: (1) multi-tiered annotation of the participants’ activities during the table-setting task (using ELAN^30^), (2) manual transcription of the participants’ speech (using ELAN), and (3) automatic transcription (using Whisper^40^, see Post-processing).

The ELAN annotation tool^30^ allows audio-visual and biosignal data to be temporally segmented and annotated across multiple hierarchically connected tiers or semantic descriptions of activities performed during trials^28^. A tier is an annotation focused on one specific aspect of the annotation, spanning the whole time course of the trial. For the purpose of EASE-specific annotations, we developed an extension to the ELAN software, named EASELan, which can be accessed via https://github.com/cognitive-systems-lab/easelan.

**Fig. 4:**
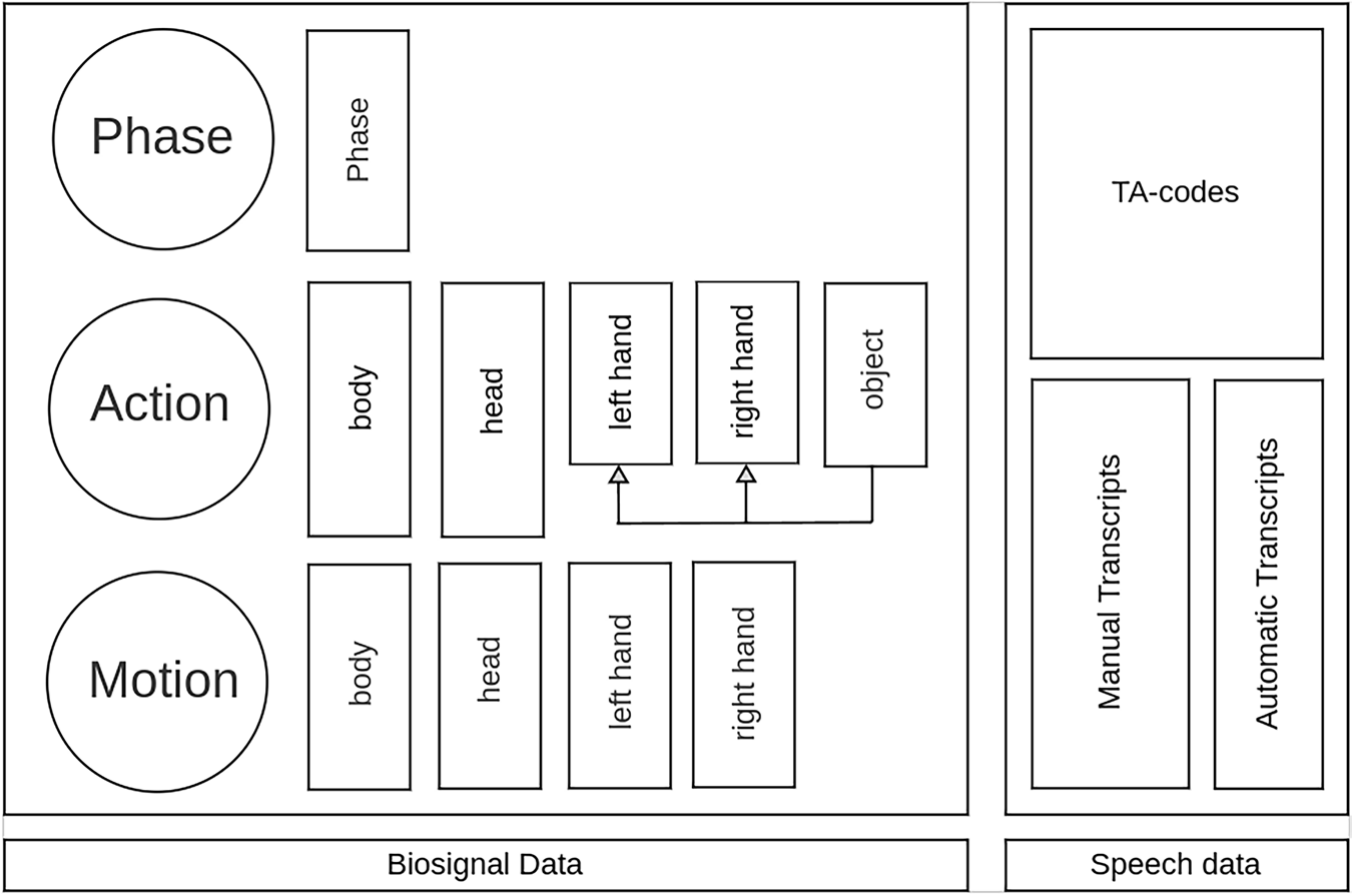
Annotation Schema: The left side shows the biosignal based annotations, the right side shows the speech data based tiers. The three levels are represented in circles while the associated tiers are in boxes.

Each table setting run is annotated on three levels of granularity. A higher level activity is *composed* of the activities from a lower level tier. However, there is usually not a one-to-many relationship since lower-level activities can span across the boundaries of two higher-level activities. The highest level contains *phase* labels, which span a sequence of *actions* in the mid-level, which again span a sequence of *motions* on the lowest level. As depicted in Fig. 4, the action and the motion level contain four tiers each for body, head, left and right hand. For each hand on the action level, there is a dependent tier containing the objects that were used in that action. The annotation schema is realized as an ELAN controlled vocabulary, which is derived from the OWL-based SOMA ontology^43^. SOMA is an open-source (LGPL) ontology used for all data collected within the EASE CRC to have consistent and semantically grounded labeling for all activities. The annotation files produced by ELAN are converted into an RDF-based semantic representation during post-processing. Thus, the EASE-TSD annotations can be joined with other datasets using SOMA-based annotations and semantically searched (e.g., using SPARQL) to find data-segments satisfying specified constraints across datasets.

For training the annotators, we provided detailed instruction documents outlining the meaning of different annotation tiers and labels. Regular workshops between annotators were conducted to discuss issues or ambigous cases. These documents are provided in the data repository and can be used to extend or modify the annotation.

Additionally, we provide automatically generated annotations of object trajectories of all kitchen objects handled in the recoreded scenes. These trajectories are mapped into the same coordinate system as the motion capture data and are extracted from knowledge-supported object tracking in the multiple RGB video streams. The detailed methodology is described in a technical report^44^.

The complexity and detail of the motion tier make annotation a difficult task as motions usually blend into each other. The annotators’s preference can also influence the duration of each annotated motion, e.g., annotating a large span or multiple smaller ones. When in doubt, annotators could add comments in a dedicated tier. Another challenge is annotating the action-hand (right/left) tier when multiple objects are involved, which demands extra attention to determine the correct alignment.

Speech transcripts compiled from think-aloud (TA) experimental trials are similarly annotated with ELAN, using a separate set of semantic rules for categorizing the vocalizations during (concurrent protocol) or after (retrospective protocol) the table-setting task^12,27,28^. These guidelines (see Table 3), a modified version of the Verbmobil transcription rules^45^, are adapted to the EASE ontology and aim to describe various cognitive processes involved in the task performance^46,47^.

**Table 3:** Think-Aloud Utterance Coding Schema.

Code	Description
Perception	Utteraces about Vision, Hearing etc.
Actions	Current/Future Actions
Plans	Describe future goal states
Methods	Talk about how they achieve a state
Issues	Indicate difficulties or confusion
Reasoning	Talk about why something occurs
Task evaluation	Talk about the state of the task
Memory	Talk about what they do (not) remember
Thoughts	Talk about thought processes
Opinions	Talk about opinions, feelings, etc.
Questions	Ask a question
Other	Talk about subjects about the task

## Data Record

The data can be accessed via the Open Science Framework platform (https://osf.io/rbyfk). The following explains the data records’ file structure, content,aggregations, and visualizations.

### File Structure

**Fig. 5:**
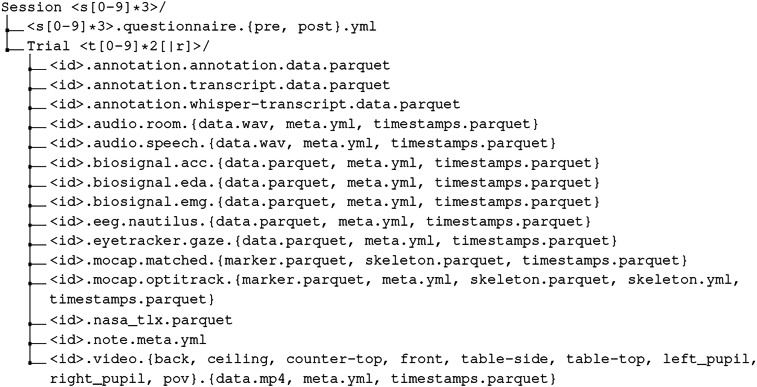
File Structure. Where <id> is the unique session and trial combination and {} indicates different file suffixes for the same base. Note that this shortens the list. When all files are present, a trial should contain 61 files.

The data is structured in hierarchical folders, where each session is a folder containing the trials, and each trial is a folder containing the data files. This structure is shown in Fig. 5. Generally, each sensor has files for data, timestamps, and meta information. The data file is sensor-specific, and the timestamps and meta file are as consistent in their format across sensors as possible. The <id> in Fig. 5 is structured as ‘s[0-9]*3-t[0-9]*2[∣r]’ where the first part is the session id and the second part is the trial id. The ‘∣r` indicates whether this is a retrospective trial, i.e., whether the participant is currently looking at herprevious trial and commenting on her actions. For example, the file ‘s049t02r.audio.speech.data.wav’ is the audio data from the retrospection of session 49 trial 2.

**Fig. 6:**
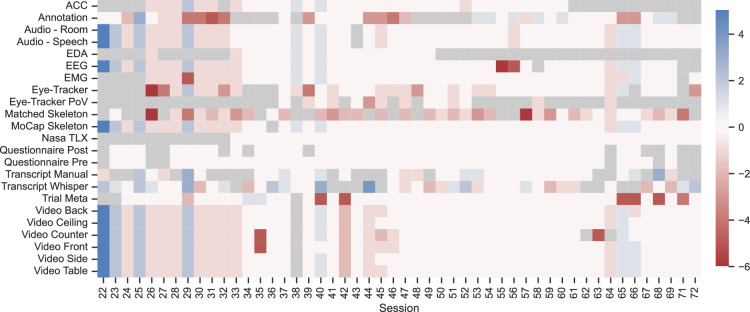
Deviation of expected files per session. The standard are six trials and one training run. Blue marks additional files, red marks less than expected and grey denotes missing files. Some participants recorded additional trials, while in other cases, trials or sensors had to be excluded for technical reasons.

Almost all sensors are fully or close to fully present across all trials and sessions. Figure 6 shows the deviation of trials per session, where a standard session is expected to include the aforementioned six trials plus one training run. As depicted, some participants agreed to record further trials, while other trials or sensor recordings had to be excluded due to technical issues. Note that sessions 63 and 64 are the only ones with the same subject. Overall, the data is balanced in terms of the number of recordings per session, with few outliers.

#### Timestamps

The timestamps file includes three columns: time, aligned, and LSL, where ”LSL" denotes the raw LSL timestamp of the sample at that time. Note that there might be a slight offset for signals relayed into LSL. This column is important for our preprocessing scripts and can mostly be ignored. The ”time" column is the relative time to the common trial beginning per sensor, e.g., the first LSL timestamp of the cut signals. The aligned column is the closest timestamp the sample in this row should belong to, adjusting for miss-timings such as signal delay, buffer queues, and other factors. The adjustment is made by redistributing timestamps based on the known or estimated sampling rate of the sensor. It can occur that two samples are assigned the same timestamp if it is unclear how to distribute them. The exact method is documented in Code Availability.

#### Meta File

The metafile includes information about the sensor, such as the channel count, channel names, sampling rate, and other sensor-specific information, such as the measured impedance, skeleton structure, or units measured. It may also include recording-specific information, such as the host, IP address, ports, and further information.

#### Transcripts

There are two types of transcripts: manual and automatic, with the latter being created using Whisper.ai (https://github.com/openai/whisper). Both are structured the same way using the begin_ts, end_ts, value, tier_name, object, and annotator columns. Begin and end give the relative timestamps from the beginning of the annotation to its end. As each sensor has a different sampling rate, the timestamp is given, and not the sample number. However, using slicing over the start and end of the timestamps’ time column, the sample numbers can be extracted, and that index is used on the data file itself (see the analysis/machine learning code in Section Code Availability for an example). The value column contains the actual annotation; the tier_name should always be a transcript, and the object should be empty. These columns are for compatibility with the other annotations. The annotator column refers to the numbered annotator id or “whisper” in case of the automatic transcript.

#### Annotations

The annotations are similarly structured to the transcripts but contain multiple tiers. The tier_name column refers to the annotation tier, e.g., motion, phase, action, etc. Special cases include the number suffix in case multiple labels should be given at the same time, e.g., if a participant is carrying multiple items with different annotation labels. In this case, the tier is duplicated. Depending on your use case, you can ignore the number suffix, e.g., if you are classifying segments or windows as in Section Technical Validation, an overlap is no issue. If you are doing sequence recognition, you might want to factor in this parallelism. Another special case is the ta-note tier, which indicates comments from the annotators. The value column follows the described annotation tier hierarchy and values as described in Methods.

#### Audio

The audio files are WAV files of each recording. The timestamp file has a slight twist: It includes a column with the samples between each entry to prevent the file from overflowing (the microphones use 41 kHz sampling rates).

#### Tabular Data

Most biosignals are stored in a tabular data format using parquet. This includes ACC, EMG, EDA, EEG, Motion Capture, and Matched Motion Capture. The columns can be read from the parquet files or their accompanying meta files’ sensor channels list. The matched motion capture is described in Methods and is the post-recording most likely matched markers and skeleton of the OptiTrack if the LSL stream omitted the skeleton due to missed settings or technical difficulties.

#### Video Data

All videos, including the 6 world views, the eye tracker views, and the pov view, are saved as mp4 files and can be read using standard video libraries.

## Data Overview

The demographics, dataset size, and exemplary visualization are outlined below. The mean age of the participants is 25.23 years (median 24 years), with a standard deviation of 5.7 years (min: 19, max: 55 years), as per the pre-study questionnaire. Twenty-two female and twenty-two male participants filled out the questionnaire (six participants did not fill out the pre-study questionnaire). 45% of participants reported to have German as native language, 18% reported English. The remainder of participants reported different native languages and chose one of the two options for conducting the experiment. 90.7% of participants reported to be right-handed. Furthermore, they reported an average table-setting experience of 3.25 (on a 5-point scale, with 1 denoting lowest level of experience) and an average experience in formal table-setting of 2.4. All answers are anonymized and provided in the dataset.

### Dataset Size

The dataset metrics are captured in Fig. 1. The dataset contains a total of eight different modalities with 22 different sensors, as well as additional information, such as questionnaires and the NASA TLX. Furthermore, transcripts and three loosely hierarchical levels splitting into ten different annotation tiers are provided. The dataset contains 50 sessions, and each session’s mean accumulated trial and retro duration is 30:18 minutes long. In total, 357 trials (738, when counting retro and test trials) were recorded, with each trial being 2:36 min long on average. The sum of all annotated activities reaches a total of 260 hours of segmented data. Note that this includes parallel annotations due to the hierarchical structure of the annotations. All sensor readings sum up to 9.8G time steps, which corresponds to a total of 300 hours of data.

**Fig. 7:**
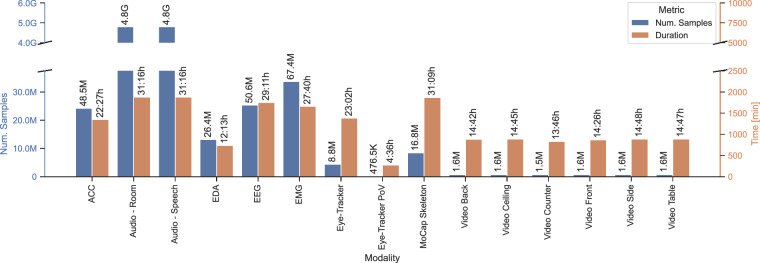
Summed sensor data. Shown as the duration in hours and the number of samples.

Figure 7 shows the summed duration and number of samples per sensor, including table setting and retrospection trials. Accordingly, data recorded during retrospection trials, such as audio and eye-tracking, have higher sums than videos. The figure also clearly shows the different sample rates affecting the number of samples with relatively similar durations. Note, furthermore, that the number of samples is not directly related to the duration, as some sample rates changed slightly over the years (further analysis in the baseline repository see Code Availability).

### Exemplary Visualization

**Fig. 8:**
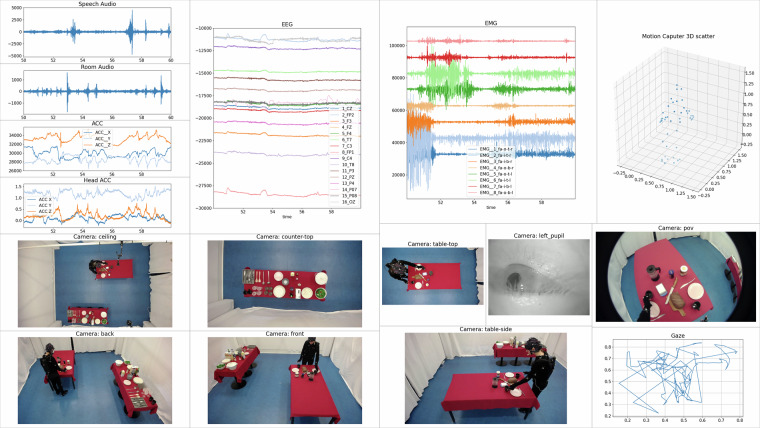
Exemplary data visualization of Session 50 Trial 02 using the authors’ LiveNodes software. From top left to bottom right: 3D OptiTrack, 2D Gaze plus tracking confidence, Nautilus EEG and ACC, PLUX EMG and ACC, near and far-field audio, six video streams. To improve legibility and resolution of the original LiveNodes output, we display a high-resolution mockup instead of the screen capture. The video is available as part of the supplementary material in the data repository.

Figure 8 shows a randomly chosen session and trial, namely Session 50, Trial 2. The plot depicts from top to bottom and left to right: the current 3D OptiTrack markers, the last ten 2D Gaze coordinates and the Pupil Core’ tracking confidence, the Nautilus last ten seconds of EEG and ACC data, the EMG-PLUX-Hubs’ and the ACC Plux-Hubs’ data, both audio streams and all six video streams. Note that in this example, the MotionCapture, video, audio, and PLUX data are without artifacts, while the EEG contains outliers, and the gaze appears quite right-sided. Both would require further post-processing and double-checking.

### Annotations

**Fig. 9:**
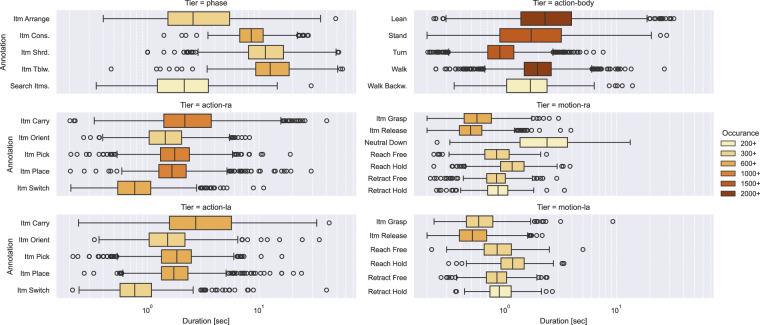
Boxplots for the duration and number of annotations per label longer than 200ms and annotated 200 times and over.

Figure 9 shows the duration and number of annotations per label longer than 200ms and annotated 200 times and over for brevity. The code is available and can be adjusted at your discretion (see Section Code Availability). The darker the boxplot color, the more annotations are present. The boxplot shows the distribution of the duration of the annotations. Please note the logarithmic time axis and different y-axes. The mean duration of the motion tier is approximately one second, while it is almost two seconds in the action tier. The action body and phase are broader and have longer and higher fluctuating mean duration. The actions lean, walk, stand, and turn are the most commonly annotated ones. Note that at the time of initial publication, not all trials have been annotated with all tiers yet and are ongoing work. The displayed number does not necessarily correlate to the number of actions in the dataset. The number of labels in each tier is roughly balanced, except for walk backward, which is annotated less than 300 times, while walk (forward) is annotated more than 2000 times.

## Technical Validation

EASE-TSD is an ongoing multi-year and pandemic-spanning work that culminates in this dataset publication. To technically validate the dataset, the previous section already presented the available files for each sensor and session, highlighting where sensors had technical issues and where they were excluded, and where they are present. Similarly, the number of samples per sensor, the mean duration and occurrences per annotated tier target, and an exemplary data plot were shown. All of this already shows a strong dataset. This section shows multiple machine learning experiments to validate the dataset further.

The ACC, EMG, EEG, and Motion Capture data were used to create a machine learning baseline for the six main tiers: phase, action-body, action-ra, action-la, motion-ra, and motion-la. The data was split using a stratified train-test group split with a 2:1 train-test ratio as typically recommended^48–50^. The split is person-independent by design, meaning that no sample of the same session is in both the training and test split at the same time. The test set comprises all data from these sessions: 25, 29, 30, 37, 43, 44, 47, 54, 60, 65 and 69. The full split is available in the code repository (https://gitlab.csl.uni-bremen.de/ease-public/tsd-one/-/blob/main/05_Analyze/baseline/split.yml).

**Fig. 10:**
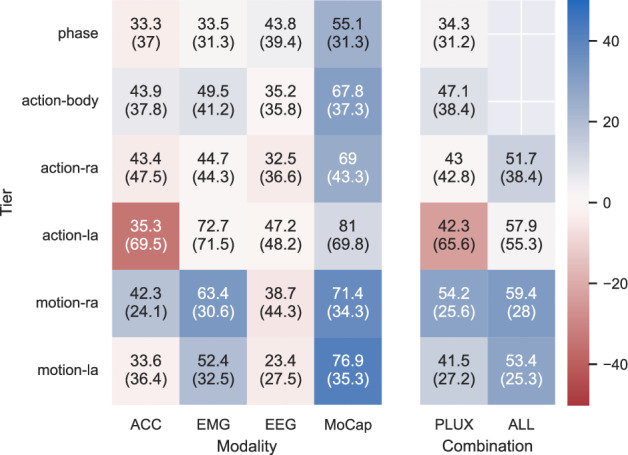
Overview of Machine Learning (ML) studies per annotation tier and modality. Each cell contains the AutoML accuracy, and the chance level in brackets below that. The cell color depicts the difference between the two, i.e., blue marks cases where the model performs better than chance level, while red marks cases in which the model underperforms. The left block uses data from single modalities. The right block combinations of modalities.

For baseline models, the data was distinguished per tier and according to the above train-test split. Each annotation segment was windowed per sensor or sensor combination. The window size aims to provide enough context for classification while not being too short. Based on Fig. 9, the window size for phase, action-body, action-ra, and action-la was set to one second, and motion-ra and motion-la were set to half a second. All shorter segments are discarded, leading to different-sized train-test splits per sensor and tier combination. Figure 10 shows the model’s performance in relation to chance level (guessing baseline). Chance level refers to the score a model only predicts the most prominent target would achieve, e.g., Scikits DummyClassifier. In each cell, the graphic gives the achieved accuracy and, in brackets, the chance level accuracy in percent of each sensor and tier combination. The left side indicates single modalities, while the right side indicates sensor combinations. PLUX refers to the combination of ACC, EMG, and EDA, which were all recorded with PLUX biosignal hubs. ALL indicates the PLUX sensors in addition to the EEG and Motion Capture. Missing entries (phase and action-body with all sensors) indicate running issues related to memory consumption on long sequences with different frequency sensors merged along the time axis. All models were created using the above-described windowing, no further preprocessing or feature extraction, and using the AutoSklearn library with a 20-thread CPU and 3 hours of search time. The AutoSklearn library does include 15 learning algorithms and 14 preprocessing methods, but no classical feature engineering methods^51^. The only exception with regard to search time is the Motion Capture, which was run initially to determine the best search time. It initially used 6 hours of search time and converged after one hour. Checking the other sensors, no accuracy over time plot indicates further learning after one hour (https://gitlab.csl.uni-bremen.de/ease-public/tsd-one/-/tree/main/06_Model/baseline/windowed/runs). The plots are available alongside the code (see Code Availability).

Figure 10 shows that some sensors are easier to learn from than others. The Motion Capture consistently outperforms the chance level, which is reasonable, as it is closest to how annotators judge each movement and includes information about all extremities. Whereas the EMG only includes information on the right hand and, therefore, performs better on that side, as shown in the motion-ra. Interestingly, this does not hold for the action target, which could indicate windows that are too short for this class and strongly encourage sequence models over window-based classification. Notably, the action-la is particularly badly predicted when using any acceleration channels, which makes sense given its location on the subject’s back but is still surprising, as at least chance level could be expected. The longer and more general tiers phase, action body, and action are generally less well recognized with local sensors, which makes sense given the short window size and sample difference. Similarly, the EEG typically requires advanced preprocessing steps in order to be predicted well. All of this indicates that the Motion Capture is the easiest to learn from all observed sensors. It can be expected that sensor-specific feature engineering, as well as sequence modeling, will improve the performance of these baseline models, and we look forward to seeing the results of these future studies.

## Data Availability

The data of 78 participants, who consented to data publication, is freely available at https://osf.io/rbyfk, accompanied by comprehensive documentation and annotation. The data is structured by session and contains seperate files for each modality and annotation type, split by trials within the session. A detailed breakdown is given in the Data Record section.

## Code availability

The study and data analysis makes extensive use of many free libraries, such as Numpy^52^, Pandas^53^, Scikit-Learn^54^, Scipy^55^, Matplotlib^56^, Seaborn^57^ and AutoSklearn^51,58^, as well as a substantial amount of custom code. Below is a list of tools created, their purpose, and where they can be accessed.

• **Nautilus Driver:** The Nautilus Driver is a custom driver for the Nautilus EEG Cap. It is used to stream the received EEG signal via LSL. The driver is written in C++ and is available on GitLab (https://gitlab.csl.uni-bremen.de/ease-public/g.nautilus-driver).

• **LSL Tools:** The LSL Tools are a set of tools to send and receive data using the Lab Streaming Layer (LSL) protocol and include simple plotting functions. The tools are written in Python and are available on GitLab (https://gitlab.csl.uni-bremen.de/ease-public/lsl-tools).

• **Experiment Control:** The Experiment Control is a custom tool to control the experiment flow. It starts and stops each trial, randomly selects the trial condition, controls lighting, retrospection videos, and more. The tool is currently under further development and is not yet available publicly.

• **Lab-Commander**: Lab-Commander (labc) is a platform-independent pure-Python zero-conf user-friendly rootless network middle-ware that originated during the setup of EASE-TSD recording architecture. It is targeted at a fast setup of short-lived distributed applications within a computer network without any prior knowledge of computer networking required (https://gitlab.csl.uni-bremen.de/ease-public/labc).

• **Processing Scripts**: The Processing Scripts are a set of Python scripts to cut all sensor data into a shared length, ensuring data presence where possible. The scripts are available on GitLab (https://gitlab.csl.uni-bremen.de/ease-public/tsd-one).

• **Machine Learning/Analysis Scripts**: The Machine Learning (ML) Scripts Analysis are a set of Marimo (http://marimo.io/) notebooks and python scripts to segment the data, train, and evaluate the models. The scripts are available on GitLab (https://gitlab.csl.uni-bremen.de/ease-public/tsd-one).
